# School bullying and social-emotional competence: the mediating role of teacher-student relationships

**DOI:** 10.3389/fpsyg.2026.1792211

**Published:** 2026-06-23

**Authors:** Li Shengxing, Song Xinyu, Xiao Wen, He Jing

**Affiliations:** 1 School of Elementary Education, Hunan First Normal University, Changsha, Hunan, China; 2 Department of Psychology, Hunan First Normal University, Changsha, Hunan, China; 3 Zhengxiang District Yanmingxi Primary School, Hengyang, Hunan, China; 4 New Era Teacher Education Innovation and Development Research Center, Changsha, Hunan, China

**Keywords:** mediation effect, preadolescents and early adolescents, school bullying, social-emotional competence, teacher-student relationships

## Abstract

**Objective:**

To investigate how both school bullying and victimization affects social–emotional competence among Chinese preadolescents and early adolescents, with a specific focus on the potential mediating effect of teacher-student relationships.

**Methods:**

The School Bullying, Social–Emotional Competence, and Teacher-Student Relationship Questionnaires were distributed to 1,760 primary and secondary school students.

**Results:**

The findings are as follows: (1) School bullying had significant negative associations with teacher-student relationships and students’ social–emotional competence. (2) Teacher-student relationships were significantly positively associated with students’ social–emotional competence. (3) Both bullying and victimization could directly affect social–emotional competence, indirectly affect social–emotional competence through teacher-student relationships (effect size was 31.35, 18.54%, respectively).

**Conclusion:**

To mitigate the negative impact of school bullying on social–emotional competence, an intervention system should be established to restore deteriorated teacher-student relationships.

## 1 Introduction

School bullying, a phenomenon defined by repeated aggressive behavior characterized by an actual or perceived imbalance of power (Olweus, 1993; Zhang, 2023), continues to represent a pervasive global concern with profound implications for adolescent development. According to a report published by United Nations Educational, Scientific and Cultural Organization (UNESCO, 2019), bullying adversely affects the mental health and overall well-being of children, severely hindering their capacity to form healthy interpersonal relationships and achieve academic success during critical developmental stages (Zhao et al., 2024). A growing body of research has identified social–emotional competence (SEC) as a key developmental domain impacted by bullying behaviors. SEC, as conceptualized by the Chinese Ministry of Education–United Nations Children’s Fund (UNICEF) Social and Emotional Learning (SEL) project team, is defined as a set of core competencies that enable students to understand and manage their relationships with themselves, others, and the collective. It comprises six dimensions: self-awareness, self-management, others-awareness, others-management, collective-awareness, and collective-management (You et al., 2023). Similarly, students who experience bullying victimization often exhibit diminished SEC, which is characterized by increased social withdrawal (Ding and Zhang, 2022). Preliminary evidence indicates that engagement in bullying is negatively associated with these competencies, potentially reflecting underlying deficits in empathy and prosocial problem-solving strategies (Graham and Juvonen, 1998; Chu et al., 2025).

The teacher–student relationship represents a critical microsystem within the educational context that may elucidate the pathway linking bullying behavior to social–emotional development (ten Bokkel et al., 2023). According to Bronfenbrenner’s (1979) ecological systems theory, the quality of proximal interactions with teachers provides students with essential emotional security and support. This, in turn serves as a foundational context for the acquisition and refinement of social–emotional skills (Deng et al., 2025). A positive teacher–student relationship—characterized by mutual warmth, trust, and minimal conflict—has been shown to be significantly associated with enhanced SEC and also serves as a protective buffer against maladaptive behavioral trajectories (Hao and Huang, 2024). Conversely, students who engage in bullying may experience strained or conflictual interactions with their teachers, thereby eroding a critical interpersonal resource that would otherwise support their own social and emotional development. While prior studies have examined the adverse consequences of bullying or the protective benefits of supportive teacher–student relationships in isolation, there exists a notable gap in the literature regarding the potential mediating role of teacher–student relationships in the specific link between bullying and victimization, and SEC. Clarifying this indirect pathway for each role is essential for the development of targeted, school-based interventions aimed at disrupting the cyclical nature of bullying and its deleterious effects.

The present study aims to address this research gap by investigating the impact of both bullying and victimization on SEC among Chinese preadolescents and early adolescents, with a specific focus on the mediating role of the teacher–student relationship. We propose the following hypotheses:

*Hypothesis 1*: Both bullying and victimization will be significantly negatively associated with SEC.

*Hypothesis 2*: Both bullying and victimization will be significantly negatively associated with the quality of teacher–student relationships.

*Hypothesis 3*: Teacher–student relationships will be significantly positively associated with SEC.

*Hypothesis 4*: The teacher–student relationship will serve as a mediator in the associations between bullying, victimization, and SEC.

To provide a more nuanced understanding of this mediating mechanism, the teacher–student relationship is further conceptualized across four distinct dimensions—intimacy, conflict, support, and satisfaction—and the differential mediating effects of each dimension will be explored for both bullying and victimization within the overall model.

Clarifying this mediating mechanism will contribute to a deeper theoretical understanding of the consequences of both bullying and victimization and will provide empirical support for the design of school-based interventions centered on improving teacher–student relationships, thereby fostering the development of SEC among preadolescents and early adolescents.

## 2 Research methodology

### 2.1 Participants and procedure

A questionnaire survey was conducted in a K–9 school (a nine-year compulsory education school) located in Hunan Province, China. Prior to data collection, ethical approval was obtained from the Institutional Review Board of the authors’ university, and the school principal provided administrative consent acting in loco parentis for this low-risk, anonymous educational survey. Data were collected between May 10, 2025 and June 11, 2025.

Students from grades 5 through 9 were invited to participate. During regular class time, researchers introduced the study’s purpose, emphasizing that participation was voluntary, anonymous, and would not affect academic records. Students provided verbal assent before completing the paper-and-pencil questionnaires. Those who declined to participate were allowed to work quietly on other tasks. No identifying information was collected.

Of the 1,761 questionnaires distributed, 1,651 were retained after removing invalid responses due to patterned answers or excessive missing data, yielding a response rate of 93.8%. The final sample comprised 912 male and 739 female students. The grade distribution was as follows: 95 fifth graders, 138 sixth graders, 633 seventh graders, 502 eighth graders, and 283 ninth graders.

Given the inclusion of upper elementary grades (grades 5 and 6), the sample is collectively referred to as preadolescents and early adolescents rather than “adolescents.” Age data were obtained via a self-report item asking students “How old are you this year (in full years)?” To protect student privacy, exact birthdates were not collected; however, students were allowed to self-report their age in years. As the sample consisted predominantly of junior high school students, the mean age based on these self-reports was 13.2 years (SD = 1.5), and the age range spanned from 10 to 16 years.

### 2.2 Research tools

#### 2.2.1 School bullying questionnaire

This study used the Olweus Bullying and Victimization Questionnaire, revised by Zhu (2005) and Hou et al. (2022). The questionnaire was structured with two dimensions: bullying and victimization. Each dimension has eight items, including physical, verbal, relational, and cyber bullying. The questionnaire uses a five-point Likert scale, with responses as follows: 1 = “never,” 2 = “less than twice a month,” 3 = “two to three times a month,” 4 = “once a week,” and 5 = “several times a week.” The bullying behavior index is represented by the total score of questions 9 through 16. The higher the total bullying behavior score, the higher the frequency of bullying behavior exhibited by the respondent; The Cronbach’s alpha coefficients for bully scale and victim scale were 0.81 and 0.74, respectively (Zhu, 2005; Ouyang, 2022).

#### 2.2.2 Social–emotional competence questionnaire

This study used a student social–emotional competence questionnaire developed by the Ministry of Education of China and UNICEF’s “Social–Emotional Learning” project team (Zhao et al., 2026). The questionnaire included six dimensions: self-awareness, self-management, others-awareness, others-management, collective-awareness, and collective-management. Each of these dimensions has five items, or a total of 30 items. The questionnaire uses a five-point Likert scale, with scores ranging from 1 (“completely disagree”) to 5 (“completely agree”). Higher total scores indicated better social–emotional competence. The Cronbach’s alpha coefficient for this scale in the present study was 0.945 (Du et al., 2018).

#### 2.2.3 Teacher-student relationships questionnaire

This study used the Teacher-Student Relationships Scale developed by Pianta and Steinberg (1992), revised by (Wang and Wang, 2022), and further revised by Zou et al. (2007) and Tan et al. (2022). The revised questionnaire consisted of 23 items divided into four dimensions: intimacy, conflict, support, and satisfaction. Among these, conflict negatively correlated with the other three dimensions; the Cronbach’s alpha coefficient was 0.87. The questionnaire uses a five-point Likert scale, with scores ranging from 1 (“completely disagree”) to 5 (“completely agree”). The Cronbach’s alpha coefficient for this scale in the present study was 0.87 (Zou et al., 2007).

### 2.3 Mathematical and statistical methods

All analyses were performed using IBM SPSS Statistics software, version 25.0. First, the collected data were screened for any common method bias. Descriptive statistics (means ± standard deviations) and Pearson’s correlation analyses were then conducted to examine the relationships among the core variables (school bullying, social–emotional competence, and teacher-student relationships). To test the hypothesized mediation model, we used Model 6 in the PROCESS macro for SPSS (Version 4.1; Hayes, 2022). The analysis utilized 5,000 bootstrap samples to estimate school bullying’s indirect effects on social–emotional competence through teacher-student relationships as the sequential mediator, while controlling for age and gender. Effects were considered statistically significant if the 95% bias-corrected confidence interval did not include zero. The significance level was set at *p* < 0.05 for all two-tailed tests. In order to run the mediation models for those children who reported significantly high scores on bullying and victimization. We use the top 50% (above the median) as the grouping criteria. This cutoff value can effectively exclude students who have no or very little experience of bullying or victimization, while retaining sufficient score variation in the independent variables to satisfy statistical assumptions in the mediation analysis. We ran all mediation models in this sub sample (n = 825, accounting for 50% of the total sample).

## 3 Results

### 3.1 Common method bias test

Common method bias was assessed using Harman’s single-factor test. The results showed that 16 factors had eigenvalues greater than 1. The first factor accounted for 31.09% of the variance, which is below the critical threshold of 40% (Podsakoff et al., 2003). Thus, common method bias was not a serious concern in this study.

### 3.2 Demographic characteristics of the sample

As shown in Table 1, 55.2% (912) of the sample were male students and 44.8% (739) were female students. Boys reported significantly higher levels of bullying than girls, and no significant difference was observed in victimization. There was no significant difference in SEC and teacher–student relationships between the genders. Significant grade-level differences were observed in school bullying, victimization, SEC, and teacher-student relationships. Specifically, mean bullying scores were highest in the 6th grade, whereas scores for victimization, SEC, and teacher-student relationships were the highest in the 9th grade among all included grade levels.

**Table 1:** Gender and grade differences (M ± SD).

Variable	Male (*n* = 912)	Female (*n* = 739)	*t*	Fifth grade (*n* = 95)	Sixth grade (*n* = 138)	Seventh grade (*n* = 633)	Eighth grade (*n* = 502)	Ninth grade (*n* = 283)	*F*
Bullying	9.58 ± 3.64	9.07 ± 3.33	2.93**	9.20 ± 2.08	9.86 ± 3.79	9.44 ± 2.87	8.94 ± 2.45	9.68 ± 5.84	3.186**
Victimization	8.60 ± 2.88	8.44 ± 2.97	1.16	8.58 ± 2,23	8.44 ± 1.69	8.36 ± 1.74	8.28 ± 1.62	9.37 ± 5.88	7.462***
Social–emotional competence	118.89 ± 19.17	119.28 ± 18.98	−0.416	113.33 ± 19.94	117.56 ± 18.21	116.52 ± 16.79	120.19 ± 17.84	125.41 ± 23.86	10.916***
Teacher-student relationships	71.94 ± 11.58	71.96 ± 11.30	−0.035	70.24 ± 10.04	70.79 ± 10.77	69.56 ± 10.16	73.89 ± 11.43	74.99 ± 13.54	16.359***

**p < 0.01, ***p < 0.001. The same below.

### 3.3 Results of the correlation analysis

As shown in Table 2, the correlation coefficients among school bullying, SEC, and teacher–student relationships were statistically significant. The results indicate negative correlations between school bullying (including bullying and victimization), teacher–student relationships, and SEC. These findings suggest that reducing school bullying and improving teacher–student relationships among preadolescents and early adolescents may help improve SEC.

**Table 2:** Variable descriptive statistics and correlation analysis.

Variable	*M ± SD*	Bullying	Victimization	Social-emotional competence	Teacher-student relationships
Bullying	9.35 ± 3.51	1			
Victimization	8.53 ± 2.92	0.737***	1		
Social-emotional competence	119.06 ± 19.08	−0.239***	−0.181***	1	
Teacher-student relationships	71.95 ± 11.45	−0.128***	−0.035***	0.564***	1

### 3.4 Analysis of mediated effects

#### 3.4.1 Teacher–student relationships

**Figure 1:**
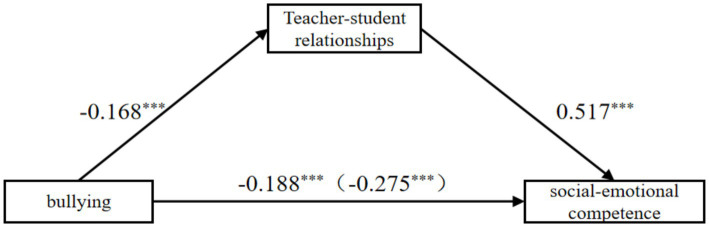
Mediated model of bullying and teacher-student relationships.

**Figure 2:**
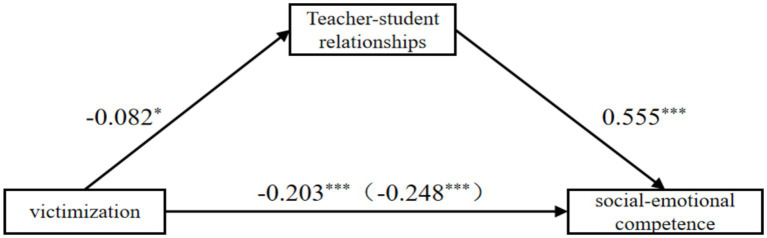
Mediated model of victimization and teacher-student relationships.

A mediation analysis test was conducted according to the non-parametric percentile bootstrap method proposed by Hayes using the PROCESS (Version 4.1) macro model 4 with a confidence interval (CI) of 95%, and controlling for age and gender, under the 5,000 bootstrap samples (Hayes, 2022). As shown in Table 3, the results showed that bullying (*β* = −0.275, *p* < 0.001) and victimization (β = −0.248, *p* < 0.001) were negatively associated with the direct path to SEC, and Hypothesis 1 was tested. Later on, the test of the mediating effect of teacher–student relationships between bullying and SEC showed that bullying (β = −0.168, *p* < 0.001) and victimization (β = −0.082, *p* < 0.05) were negatively associated with teacher–student relationships, and Hypothesis 2 was tested. Finally, the teacher–student relationships were taken as an intermediary variable, and the negative association effects of bullying (β = −0.188, *p* < 0.001) and victimization (β = −0.203, *p* < 0.001) on SEC remained significant (Figures 1, 2).

**Table 3:** Mediation effect test of teacher-student relationships on bullying and SEC.

Variable	Type of effect	Efficiency value	Boot *SE*	(Boot)95%CI		Proportion of total effect
				Boot LLCI	Boot ULCI	
Bullying	Total effect	−1.18	0.15	−1.47	−0.88	100%
	Direct effect	−0.80	0.13	−1.06	−0.55	68.65%
	Mediated effect	−0.37	0.14	−0.65	−0.17	31.35%
Victimization	Total effect	−1.24	0.17	−1.58	−0.90	100%
	Direct effect	−1.01	0.14	−1.30	−0.73	81.46%
	Mediated effect	−0.23	0.20	−0.62	−0.17	18.54%

The results of the mediation effect analysis indicated that the mediated effect of bullying is −0.37, with a 95% CI of [−0.65, −0.17], and the mediated effect of victimization is −0.23, with a 95% CI of [−0.62, −0.17] (Table 3). These results suggest that the mediating effect of teacher–student relationships is significant in the impact of bullying and victimization on SEC (effect size was 31.35, 18.54%, respectively).

#### 3.4.2 The impact of various dimensions of teacher–student relationships

Teacher–student relationships were conceptualized into four dimensions: intimacy, conflict, support, and satisfaction. The present study also explored the mediating effects of different dimensions.

##### 3.4.2.1 Intimacy

**Figure 3:**
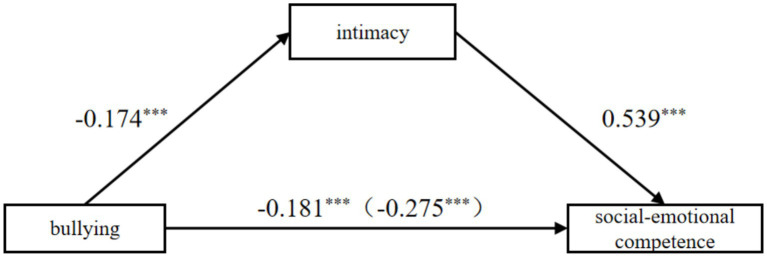
Mediated model of bullying and intimacy.

**Figure 4:**
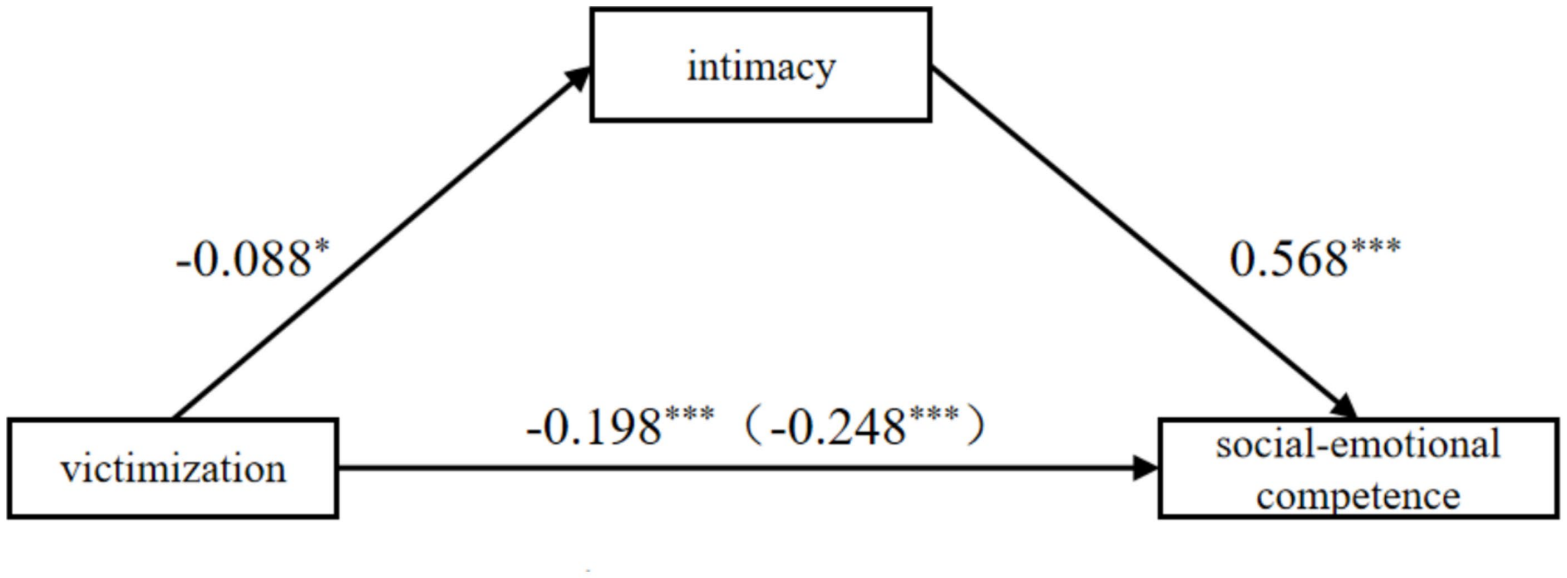
Mediated model of victimization and intimacy.

Regression analysis results showed that bullying (*β* = −0.275, *p* < 0.001) and victimization (*β* = −0.248, *p* < 0.01) were significantly negatively associated with SEC. Bullying (β = −0.174, *p* < 0.001) and victimization (*β* = −0.088, *p* < 0.05) were also negatively associated with intimacy. After adding intimacy as a mediating variable, bullying (*β* = −0.181, *p* < 0.001) and victimization (β = −0.198, *p* < 0.001) were also negatively associated with SEC (Figures 3, 4). The results of the mediation analysis indicated that the mediated effect of bullying is −0.40, with a 95% CI of [−0.63, −0.19], and the mediated effect of victimization is −0.25, with a 95% CI of [−0.54, −0.04] (Table 4). These results suggest that the mediating effect of intimacy is significant in the impact of bullying and victimization on SEC (effect size was 33.89, 20.16%, respectively).

**Table 4:** Mediation effect test of intimacy on school bullying and social–emotional competence.

Variable	Type of effect	Efficiency value	Boot *SE*	(Boot)95%CI		Proportion of total effect
				Boot LLCI	Boot ULCI	
Bullying	Total effect	−1.18	0.15	−1.47	−0.88	100%
	Direct effect	−0.78	0.13	−1.03	−0.53	66.11%
	Mediated effect	−0.40	0.12	−0.63	−0.19	33.89%
Victimization	Total effect	−1.24	0.17	−1.58	−0.90	100%
	Direct effect	−0.99	0.14	−1.27	−0.71	79.84%
	Mediated effect	−0.25	0.15	−0.54	−0.04	20.16%

##### 3.4.2.2 Conflict

**Figure 5:**
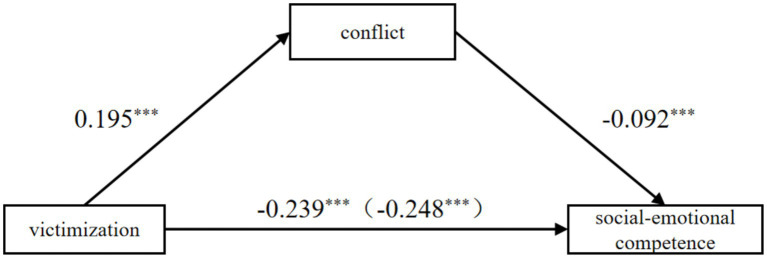
Mediated model of victimization and conflict.

Regression analysis results showed that victimization (*β* = −0.248, *p* < 0.001) was significantly negatively associated with social–emotional competence. Victimization (*β* = −0.195, *p* < 0.001) was also associated with conflict. After adding conflict as a mediating variable, victimization (β = −0.239, *p* < 0.001) was also negatively associated with SEC (Figure 5). The results of the mediation analysis indicate that the mediated effect of victimization is −0.09, with a 95% CI of [−0.24, −0.01] (Table 5). These results suggest that the conflict significantly mediates the relationship between victimization and SEC (effect size was 7.25%).

**Table 5:** Mediation effect test of conflict on school bullying and social–emotional competence.

Variable	Type of effect	Efficiency value	Boot *SE*	(Boot)95%CI		Proportion of total effect
				Boot LLCI	Boot ULCI	
Victimization	Total effect	−1.24	0.17	−1.58	−0.90	100%
	Direct effect	−1.15	0.18	−1.50	−0.81	92.75%
	Mediated effect	−0.09	0.06	−0.24	−0.01	7.25%

##### 3.4.2.3 Support

**Figure 6:**
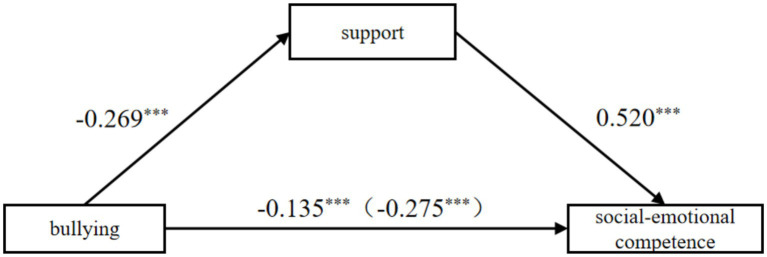
Mediated model of bullying and support.

**Figure 7:**
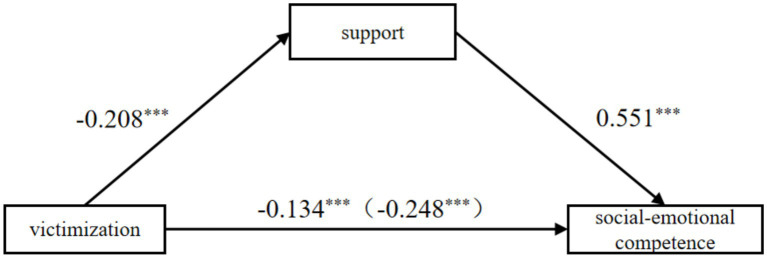
Mediated model of victimization and support.

Regression analysis results showed that bullying (*β* = −0.275, *p* < 0.001) and victimization (*β* = −0.134, *p* < 0.001) were significantly negatively associated with SEC. Bullying (β = −0.269, *p* < 0.001) and victimization (*β* = −0.208, *p* < 0.001) were also negatively associated with support. After adding support as a mediating variable, bullying (*β* = −0.135, *p* < 0.001) and victimization (β = −0.134, *p* < 0.001) were also negatively associated with SEC (Figures 6, 7). The results of the mediation analysis indicate that the mediated effect of bullying is −0.60, with a 95% CI of [−0.82, −0.39], and the mediated effect of victimization is −0.57, with a 95% CI of [−0.88, −0.29] (Table 6). These results suggest that the mediating effect of support is significant in the impact of school bullying on SEC (effect size was 50.84, 45.96%, respectively).

**Table 6:** Mediation effect test of support on school bullying and social–emotional competence.

Variable	Type of effect	Efficiency value	Boot *SE*	(Boot)95%CI		Proportion of Total Effect
				Boot LLCI	Boot ULCI	
Bullying	Total effect	−1.18	0.15	−1.47	−0.88	100%
	Direct effect	−0.58	0.13	−0.84	−0.32	49.16%
	Mediated effect	−0.60	0.11	−0.82	−0.39	50.84%
Victimization	Total effect	−1.24	0.17	−1.58	−0.90	100%
	Direct effect	−0.67	0.15	−0.96	−0.38	54.04%
	Mediated effect	−0.57	0.14	−0.88	−0.29	45.96%

##### 3.4.2.4 Satisfaction

**Figure 8:**
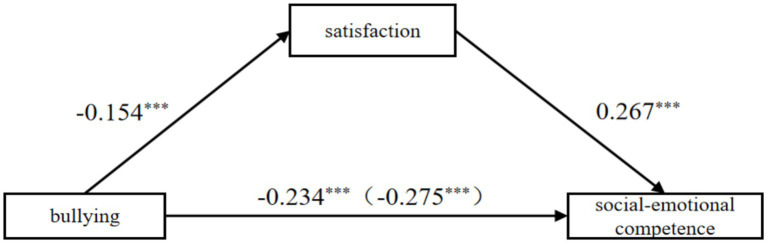
Mediated model of bullying and satisfaction.

**Figure 9:**
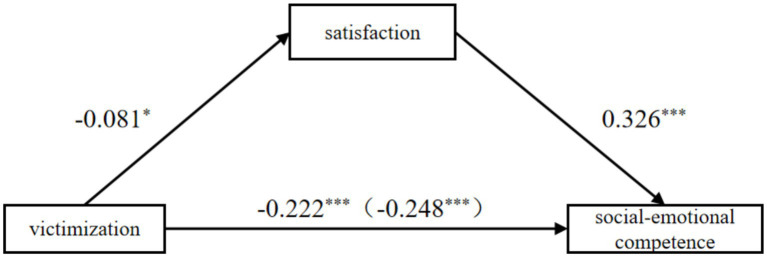
Mediated model of victimization and satisfaction.

Regression analysis results show that bullying (β = −0.275, *p* < 0.001) and victimization (β = −0.248, *p* < 0.001) were significantly negatively associated with SEC. Bullying (β = −0.154, *p* < 0.001) and victimization (β = −0.081, *p* < 0.05) were also negatively associated with satisfaction. After adding satisfaction as a mediating variable, bullying (β = −0.234, *p* < 0.001) and victimization (β = −0.222, *p* < 0.001) were also negatively associated with SEC (Figures 8, 9). The results of the mediation analysis indicate that the mediated effect of bullying is −0.18, with a 95% CI of [−0.28, −0.09], and the mediated effect of victimization is −0.13, with a 95% CI of [−0.27, −0.03] (Table 7). These results suggest that the mediating effect of satisfaction is significant in the impact of school bullying on SEC (effect size was 15.25, 10.48%, respectively).

**Table 7:** Mediation effect test of satisfaction on school bullying and social–emotional competence.

Variable	Type of effect	Efficiency value	Boot *SE*	(Boot)95%CI		Proportion of total effect
				Boot LLCI	Boot ULCI	
Bullying	Total effect	−1.18	0.15	−1.47	−0.88	100%
	Direct effect	−1.00	0.15	−1.29	−0.72	84.75%
	Mediated effect	−0.18	0.05	−0.28	−0.09	15.25%
Victimization	Total effect	−1.24	0.17	−1.58	−0.90	100%
	Direct effect	−1.11	0.17	−1.43	−0.79	89.52%
	Mediated effect	−0.13	0.06	−0.27	−0.03	10.48%

## 4 Discussion

### 4.1 Summary and interpretation of key findings

#### 4.1.1 The significant negative association between both bullying and victimization and students’ SEC

This study’s findings indicate that bullying and victimization were significantly negatively associated with students’ SEC, supporting H1. This result is consistent with that of previous studies.

For students who experience victimization, bullying victimization not only directly impairs students’ ability to regulate emotions and engage in social interactions but also further undermines their social–emotional functioning by triggering internalizing symptoms, such as anxiety and depression; externalizing symptoms, such as aggressive behavior and attention problems; and alexithymia (Arseneault et al., 2010; Prino et al., 2019; Trompeter et al., 2024). According to the general strain theory, bullying as a persistent psychosocial stressor elicits negative emotional states, hindering students from developing adaptive emotional management and interpersonal strategies (Agnew, 1992; Agnew and Brezina, 2019; Lee et al., 2022). A comprehensive meta-analysis by Mullan et al. (2023) demonstrated a stable and significant, moderately positive correlation between peer victimization and higher levels of internalizing symptoms (e.g., depression and anxiety). Emotional distress, social withdrawal, and negative self-perceptions, characterized by internalizing symptoms, are direct manifestations of impairment in the core domains of social–emotional competencies, namely, self-management, self-awareness, and social awareness (Colomeischi et al., 2022). As a persistent, negative interpersonal interaction, bullying directly erodes victims’ ability to regulate emotions, leaving them in a prolonged state of stress, fear, and sadness, and hampering effective self-management (Vacca et al., 2023). Simultaneously, the negative messages of “rejection” and “worthlessness” conveyed by bullying experiences severely damage individuals’ self-awareness and distort their self-perception (Wang et al., 2023). This finding is consistent with the significant, negative correlation between bullying and low self-esteem revealed in the previously discussed meta-analysis (Mullan et al., 2023). Low self-esteem further weakens an individual’s confidence in and ability to establish healthy interpersonal relationships and affects the development of social awareness and relationship skills (Camodeca and Goossens, 2005; Mullan et al., 2023).

For students who engage in bullying perpetration, social–emotional deficits are primarily evident in empathy and behavior regulation. Meta-analytic evidence indicates that bullies show significant deficits in affective and cognitive empathy, reflecting impairments in other-cognition and other-management competencies (Imuta et al., 2022). Furthermore, these students often exhibit poor emotion regulation and impulse control, along with moral disengagement that weakens collective-cognition and prosocial behavior (Gini et al., 2014; Luo and Bussey, 2023).

#### 4.1.2 The significant negative association between both bullying and victimization and teacher–student relationships

This study’s findings indicate that both bullying and victimization were significantly negatively associated with intimacy in student-teacher relationships and positively associated with conflict, supporting H2. This result is consistent with that of previous studies (Elledge et al., 2016; Demol et al., 2020; Krause and Smith, 2023). However, the mechanisms underlying these associations may differ for perpetration and victimization.

For students who experience victimization, the negative association with teacher–student relationships can be explained by the psychological and behavioral changes observed in bullied students (Krause and Smith, 2023). Bullied students frequently exhibit social withdrawal and deficits in social skills (Fox and Boulton, 2005; Ding and Zhang, 2022), which may lead them to proactively reduce interactions with teachers and create emotional distance and estrangement in the student-teacher relationship. However, according to the social information-processing model (Crick and Dodge, 1994), victimization experiences can predispose individuals to discern hostile intent in others’ actions (Camodeca and Goossens, 2005; Kellij et al., 2022). Consequently, these students may be more inclined to perceive teachers’ criticism or routine disciplinary actions as hostile and unjust, which can exacerbate their perceptions of conflict within the student-teacher relationship. Therefore, school bullying not only directly undermines students’ psychological well-being but may also compromise the teacher-student relationship as one of their most crucial protective relationships within the school context.

For students who engage in bullying perpetration, the erosion of teacher–student relationships may operate through different pathways. Students who bully others often exhibit externalizing behaviors, oppositional tendencies, and poor behavioral regulation (Marengo et al., 2018; Grigore and Di Norcia, 2025). Additionally, bullies tend to show lower levels of empathy and prosocial behavior (Imuta et al., 2022). From the perspective of the social-information processing model, aggressive children tend to attribute hostile intent to the actions of peers and authority figures (Crick and Dodge, 1994; Nault and Morningstar, 2025), which may similarly color their perceptions of teacher behavior, leading them to interpret even neutral teacher interactions as confrontational and thereby intensifying perceived conflict.

#### 4.1.3 The significant positive association between teacher–student relationships and students’ SEC

This study’s findings indicate that teacher-student relationships were significantly positively associated with students’ social–emotional competence, supporting H3. This result is consistent with that of previous studies (Li et al., 2021; Chen et al., 2024; Magro et al., 2024). A meta-analysis of nearly 30,000 students confirmed that teacher-student relationships quality is systematically linked to social competence and that this association remains consistent across different educational stages (Magro et al., 2024). From the ecological systems theory perspective, the teacher-student relationships functions as a “proximal process” within the microsystem, directly driving students’ socioemotional development (Bronfenbrenner and Ceci, 1994; Deng et al., 2025). A positive teacher-student relationships provides students with emotional support and psychological security, which creates favorable conditions to develop their SEC (Di Lisio et al., 2025). Specifically, intimacy and support within this relationship foster students’ trust in their teachers, which then encourages them to more proactively seek help and guidance, facilitating progress in emotional management, self-awareness, and social awareness (Li et al., 2021; Zhang et al., 2024). For instance, a study by Li et al. (2021) demonstrated that teachers’ SEC indirectly mediates that of students, highlighting the bridging function of such relationships in emotional education (Rauterkus et al., 2025). Chen et al. (2024) further demonstrated that teacher-student interactions are more strongly associated with SEC at the school level than individually; this suggests that the overall climate of teacher-student interactions within a school is critical for cultivating students’ social–emotional development, and particularly in such areas as collective cognition. Therefore, strengthening the teacher-student relationships not only buffer the negative impacts of school bullying but also directly enhances students’ SEC, making it an indispensable protective factor in education.

#### 4.1.4 Teacher–student relationships mediate the relationship between school bullying and SEC

This study’s results indicate that teacher–student relationships significantly mediated the association between bullying and victimization and students’ SEC. Further analysis of the current research revealed that bullying and victimization indirectly impairs students’ social–emotional competence by undermining intimacy, support and satisfaction in teacher-student relationships. Simultaneously, victimization is positively associated with conflicting teacher-student relationship, exacerbating its detrimental relationship with SEC. These findings support H4.

From a theoretical perspective, social-ecological theory posits that the teacher-student relationship, as a central component of the adolescent school microsystem, directly shapes an individual’s social–emotional development through the quality of its interactions (Hong and Espelage, 2012; Yuan and Yang, 2025). This research indicates that teacher-student relationships are critical in the association between school bullying and SEC, and that they function through a dual-path mechanism. Intimacy and support teacher-student relationships are protective factors that directly mitigate the psychological distress caused by bullying through the provision of emotional warmth, trust, and security (Healy et al., 2024). Simultaneously, these relationships create a safe developmental context conducive to the acquisition of key social–emotional abilities, including empathy and emotional regulation (Sulkowski and Simmons, 2018; ten Bokkel et al., 2023). However, as destructive interpersonal trauma, bullying directly erodes students’ positive perceptions of teacher-student relationships (Forsberg et al., 2024). As evidenced by Marengo et al. (2018), victimized students are more likely to develop conflicts with teachers, often interpreting teacher-student interactions as indifferent or unfair (Jiang et al., 2024). This perception diminishes their satisfaction with and sense of support for these relationships and deprives them of the critical external resources to learn adaptive social and emotional regulatory skills (Wu and Zhang, 2022). Consequently, the pathway through which positive teacher-student interactions foster social–emotional development is disrupted, ultimately exacerbating the competency deficits resulting from bullying (Marengo et al., 2018; Thornberg et al., 2025).

Notably, the present study found that among the four dimensions of teacher-student relationships, the support dimension demonstrated the most pronounced mediating effect in the association between school bullying/victimization and SEC. This finding corroborates the conclusions of Tang (2025) and Ye (2023), who reported that supportive teacher-student relationships exert the strongest influence on students’ SEC. Theoretically, when students experience bullying, their fundamental psychological needs for safety, belonging, and competence are directly threatened. Supportive teacher-student relationships—characterized by teachers’ willingness to help, fair treatment, positive regard, and encouragement—can directly counteract this threat. As Rucinski et al. (2017) argued, for children whose self-concepts are still developing, the perceived quality of personalized support has a decisive impact on their developmental trajectories. Such support acts as a secure base, providing bullied students with reliable comfort and problem-solving assistance, thereby buffering the erosion of their social–emotional competence. Practically, this implies that teachers cannot rely solely on creating a warm general classroom climate; rather, they must proactively ensure that every student—especially those at risk of bullying—personally experiences individualized care and reliable help from the teacher. Thus, the support dimension represents the most critical leverage point in breaking the pathway from bullying to deficits in SEC.

At the class level, Thornberg et al. (2018) further demonstrated that teacher-student conflicts fail to provide effective social support for students, and the signals of rejection they convey may also intensify students’ isolation within the peer group (Krause and Smith, 2023); this is an additional source of stress that impedes the development of their SEC (Thornberg et al., 2018; Zhou et al., 2026). Taken together, the above evidence refines the differential roles in teacher-student relationships dimensions: positive dimensions (satisfaction, support, and intimacy) buffer against school bullying’s impact on SEC, while the conflict dimension acts as an exacerbating amplifier intensifying adverse effects.

By deconstructing the multidimensional mediating mechanism of teacher-student relationships, this study elucidates the social-ecological theory’s micro-level role in explaining the association between school bullying and SEC. It also transcends the previous overgeneralized understanding of this mediating effect. The methodology provides a dual-path intervention approach for targeted anti-bullying interventions—enhancing positive teacher-student interactions while resolving relational conflicts—thus highlighting the unique value of targeted dimensional interventions in fostering SEC among bullied students.

However, the above finding of partial mediation simultaneously suggests that relying solely on improving teacher–student relationships—a relational pathway—are insufficient to fully buffer the negative impact of bullying on SEC. Consequently, future intervention efforts should integrate “enhancing the quality of teacher training” with “scaling up child- and classroom-focused social–emotional learning (SEL) curricula” to build a more comprehensive protective system. Internationally, multiple evidence-based SEL programs have been widely implemented with demonstrable effectiveness: The Collaborative for Academic, Social, and Emotional Learning (CASEL) framework, which delineates five core competencies—self-awareness, self-management, social awareness, relationship skills, and responsible decision-making—together with CASEL’s “SEL 3 Signature Practices,” provides teachers with accessible, classroom-embedded strategies that require no standalone curriculum (CASEL, 2020). In a significant 2024 update, its program guide expanded to include 99 programs (CASEL, 2024). The RULER approach, developed at the Yale Center for Emotional Intelligence, systematically cultivates emotional intelligence from pre-kindergarten through high school (Brackett et al., 2019). The SEE Learning curriculum from Emory University has not only gained “SELect” status but has seen successful implementation in East Asia, with a 2024 study in South Korea demonstrating significant improvements in student resilience (Min et al., 2024). The National Youth Social and Emotional Competence Thousand-School Alliance, initiated by East China Normal University in 2023, has kept expanding its member schools and promoted the localized practice of SEL in China’s education system (East China Normal University, 2023).

The curriculum-based intervention strategies described above directly cultivate students’ emotional cognition, empathy, and conflict-resolution skills through structured classroom activities, complementing and synergizing with the relational support afforded by positive teacher–student interactions. Accordingly, this study recommends that schools, while striving to enhance the quality of teacher–student relationships, should also systematically introduce and implement developmentally appropriate SEL curricula, thereby leveraging coordinated efforts at both the relational and curricular levels to promote adolescents’ SEC more comprehensively and sustainably.

### 4.2 Comparison with recent studies

Recent studies since 2022 have further corroborated the relational mechanisms linking social–emotional factors and bullying. Ma and Niu (2025) found that teachers’ SEC affects bullying in rural Chinese schools through the chain mediation of students’ SEC and classmate relationships. You et al. (2023) similarly demonstrated that SEL reduces bullying via the serial mediation of SEC and peer relationships. Yang (2025) extended this to student engagement, with teacher–student relations, peer relations, and school identification as collective mediators. Most recently, Li et al. (2026), using the Organization for Economic Co-operation and Development’s Survey on Social and Emotional Skills data from Suzhou, showed that perceived relationships with teachers and school belonging significantly mediated the link between social–emotional skills and bullying. Notably, their finding that the negative association was substantially reduced but not eliminated when relational factors were accounted for is consistent with the present study’s core finding of partial mediation. While prior studies have relied on global measures of teacher–student relationships, the present study disaggregates this construct into four dimensions—intimacy, conflict, support, and satisfaction—offering a more fine-grained analysis. Moreover, unlike investigations centered on social–emotional predictors or victimization, the current study positions student bullying behavior as the independent variable and SEC as the outcome, with teacher–student relationships as the key mediator.

### 4.3 Limitations and future research directions

Several limitations of the present study should be acknowledged, each of which points to meaningful directions for future research.

First, this study employed a cross-sectional design, which precludes causal inference regarding the mediating role of teacher-student relationships. Maxwell and Cole (2007) warned that cross-sectional mediation analyses tend to produce biased estimations of longitudinal parameters. This methodological limitation is prominent in studies investigating constructs with dynamic reciprocal associations across time, such as bullying behavior, teacher-student relationships, and SEC. Despite this well-recognized concern, a 2025 scoping review of quantitative evidence on mediating and moderating mechanisms between social media use and adolescent aggression found that cross-sectional designs predominated among the 44 studies reviewed (Giannakopoulos and Prassou, 2025). Future research should adopt longitudinal or experimental designs to establish the causal sequencing implied in our mediation model.

Second, the sample generalizability of this study is constrained by its geographic focus on a single K–9 school in Hunan Province, China. Cross-cultural research has long noted that findings on SEC in one cultural-educational context may not directly translate to others (Chen and French, 2008). Recent research has further revealed substantial heterogeneity in bullying prevalence across Chinese educational contexts. A 2025 study found that bullying victimization in rural Chinese primary schools was highly prevalent, and noted that existing research has been concentrated largely in urban areas (Liu et al., 2025). Meanwhile, a 2024 investigation in Qingyang City documented a 47.35% bullying reporting rate, with higher incidence among students in urban–rural integration junior high schools compared to those in municipal-level schools (Li et al., 2024). Future research should recruit larger, more diverse samples across multiple regions and school types to enhance the external validity of these findings.

## 5 Conclusion

This study examined the relationships among school bullying, SEC, and teacher–student relationships in a sample of Chinese preadolescents and early adolescents. The results supported all four hypotheses: Student bullying behavior was significantly and negatively associated with both SEC and teacher–student relationships; teacher–student relationships were significantly and positively associated with SEC; and, most critically, teacher–student relationships partially mediated the association between bullying behavior and SEC. By disaggregating the teacher–student relationship into four distinct dimensions—intimacy, conflict, support, and satisfaction—this study further revealed the differential mediating effects of each dimension, thereby providing a more fine-grained understanding of this relational mechanism.

The finding of partial rather than full mediation carries important practical implications: Improving teacher–student relationships can buffer, but not entirely offset, the negative impact of bullying on adolescents’ social–emotional development. Accordingly, school-based intervention efforts should integrate both relational strategies—such as enhancing teacher–student interactions and restoring deteriorated relationships—and curricular approaches—such as systematic SEL programs—to more comprehensively support students’ SEC.

## Data Availability

The raw data supporting the conclusions of this article will be made available by the authors, without undue reservation.
